# Effect of Low-Protein Diet and Inulin on Microbiota and Clinical Parameters in Patients with Chronic Kidney Disease

**DOI:** 10.3390/nu11123006

**Published:** 2019-12-09

**Authors:** Silvia Lai, Alessio Molfino, Massimo Testorio, Adolfo M. Perrotta, Annachiara Currado, Giovanni Pintus, Daniele Pietrucci, Valeria Unida, Davide La Rocca, Silvia Biocca, Alessandro Desideri

**Affiliations:** 1 Department of Translational and Precision Medicine, Sapienza University of Rome, 00185 Rome, Italy; alessio.molfino@uniroma1.it (A.M.); adolfo.perrotta@gmail.com (A.M.P.); annachiaracurrado@gmail.com (A.C.); pintusgiovanni@hotmail.it (G.P.); 2 Department of Obstetrical-Gynecological Sciences and Urologic Sciences, Unit of Nephrology, Sapienza University of Rome, 00185 Rome, Italy; massimo.testorio@uniroma1.it; 3 Department of Biology, Tor Vergata University of Rome, 00133 Rome, Italy; daniele.pietrucci@uniroma2.it (D.P.); desideri@uniroma2.it (A.D.); 4 Department of Systems Medicine, Tor Vergata University of Rome, 00133 Rome, Italy

**Keywords:** microbiota, prebiotic therapy, chronic kidney disease, low-protein diet, inulin, endothelial dysfunction

## Abstract

Introduction: The gut microbiota has coevolved with humans for a mutually beneficial coexistence and plays an important role in health and disease. A dysbiotic gut microbiome may contribute to progression to chronic kidney disease (CKD) and CKD-related complications such as cardiovascular disease. Microbiota modulation through the administration of prebiotics may represent an important therapeutic target. Aim: We sought to evaluate the effects of a low-protein diet (LPD) (0.6 g/kg/day) with or without the intake of the prebiotic inulin (19 g/day) on microbiota and clinical parameters in CKD patients. Materials and Methods: We performed a longitudinal, prospective, controlled, and interventional study on 16 patients: 9 patients treated with LPD (0.6 g/kg/day) and inulin (19 g/day) and 7 patients (control group) treated only with LPD (0.6 g/kg/day). Clinical evaluations were performed and fecal samples were collected for a subsequent evaluation of the intestinal microbiota in all patients. These tests were carried out before the initiation of LPD, with or without inulin, at baseline (T0) and at 6 months (T2). The microbiota of 16 healthy control (HC) subjects was also analyzed in order to identify potential dysbiosis between patients and healthy subjects. Results: Gut microbiota of CKD patients was different from that of healthy controls. The LPD was able to significantly increase the frequencies of Akkermansiaceae and Bacteroidaceae and decrease the frequencies of Christensenellaceae, Clostridiaceae, Lactobacillaceae, and Pasteurellaceae. Only Bifidobacteriaceae were increased when the LPD was accompanied by oral inulin intake. We showed a significant reduction of serum uric acid (SUA) and C-reactive protein (CRP) in patients treated with LPD and inulin (*p* = 0.018 and *p* = 0.003, respectively), an improvement in SF-36 (physical role functioning and general health perceptions; *p* = 0.03 and *p* = 0.01, respectively), and a significant increase of serum bicarbonate both in patients treated with LPD (*p* = 0.026) or with LPD and inulin (*p* = 0.01). Moreover, in patients treated with LPD and inulin, we observed a significant reduction in circulating tumor necrosis factor alpha (TNF-α) (*p* = 0.041) and plasma nicotinamide adenine dinucleotide phosphate (NADPH) oxidase (NOX2) (*p* = 0.027) levels. We did not find a significant difference in the circulating levels of Interleukin (IL)-1β (*p* = 0.529) and IL-6 (*p* = 0.828) in the two groups. Conclusions: LPD, associated or not with inulin, modified gut microbiota and modulated inflammatory and metabolic parameters in patients with CKD. Our results suggest that interventions attempting to modulate the gut microbiome may represent novel strategies to improve clinical outcomes in CKD patients and may provide useful therapeutic effects.

## 1. Introduction

Normal gut microbiota influences the well-being of the host by contributing to its nutrition, metabolism, physiology, and immune function. Disturbance of normal gut microbiota (dysbiosis) has been implicated in the pathogenesis of diverse clinical conditions, such as obesity [1], type 2 diabetes [2], inflammatory bowel disease [3], and cardiovascular [4] and neurological diseases [5]. Quantitative and qualitative alterations in gut microbiota are noted in patients with chronic kidney disease (CKD) and end-stage renal disease (ESRD) [6]. Preliminary evidence indicates that toxic products generated by a dysbiotic gut microbiome may contribute to progression to CKD and CKD-related complications, such as cardiovascular disease [6]. CKD patients display endothelial dysfunction characterized by an impairment of endothelium-dependent vasodilation and an increase in plasma levels of endothelial molecules involved in vascular tone, hemostasis, or adhesion. Endothelial dysfunction plays an important role in the development of cardiovascular diseases, which are the leading cause of mortality in CKD patients [7]. Systemic inflammation, accompanied by oxidative stress in humans and animals, is invariably present and plays a major role in the progression to ESRD and cardiovascular risk. Several factors contribute to the pathogenesis of CKD-associated oxidative stress and inflammation, including profound changes in the composition and function of the intestinal microbiome and disruption of the gut epithelial barrier, a structure leading to generation and influx of endotoxins, microbial fragments, and other toxic and proinflammatory products in the body’s internal milieu [8]. In this light, studies have shown marked disintegration of the colonic epithelial barrier structure and significant alteration of the colonic bacterial flora in humans and animals with advanced CKD, as well as the disruption of the normal symbiotic relationship and production, absorption, and retention of toxic products [6,7]. The alteration of the microbial flora can contribute to systemic inflammation, endothelial dysfunction, and uremic toxicity [8]. Microbiota could represent an important therapeutic target that can likely be modulated by administering a low-protein diet (LPD), which has been shown to reduce uremic toxin production by the gut microbiota in nondialysis CKD patients, and potentially by prebiotics, which are nondigestible organic substances that can be fermented by the host, leading to beneficial effects in the host [9]. In particular, inulin, which is already present in some aproteic foods, has a neutral taste and limited side effects and has been shown to increase the growth of bifidobacteria and decrease that of pathogenic bacteria [10].

In this study, we evaluated the effects of LPD (0.6 g/kg/day), with or without the intake of the prebiotic inulin (19 g/day), on microbiota and clinical parameters in CKD patients.

## 2. Materials and Methods

The study protocol was approved by the Local Clinical Research Ethics Committee with protocol number 302/17, 4465, and we obtained the written consent of each patient enrolled.

### 2.1. Study Design and Subjects

We performed an interventional, prospective, and controlled study on 16 patients—9 patients treated with LPD (0.6 g/kg/day) and inulin (19 g/day) and 7 controls group treated only with LPD (0.6 g/kg/day) without inulin—who were matched by sex and estimated glomerular filtration rate (eGFR) at the Hospital “Policlinico Umberto I” of Rome, Sapienza University of Rome, Italy. Patients were enrolled from June 2017 to January 2018. All patients had never previously undergone diet therapy. The LPD was developed by an expert dietitian who prescribed a personalized nutritional therapy for each patient of low protein intake and high biological value, including protein-free food products, preferring base-inducing proteins by fruit and vegetable intake, with a low concentration of salt, phosphorus, potassium, and acid-inducing dietary proteins but an adequate caloric intake (30–35 kcal/kg/day). Adherence to the diet during the visits was verified every 3 months through the evaluation of urinary nitrogen. Clinical and biochemical evaluations were performed and fecal samples were collected for a subsequent evaluation of the intestinal microbiota in all patients. These tests were carried out before starting the LPD with or without inulin, at baseline (T0), and at 6 months (T1). The microbiota of 16 healthy control (HC) subjects was also analyzed in order to identify potential dysbiosis between patients and healthy subjects. 

### 2.2. Patients

We enrolled patients with CKD stage 3G–4G Kidney Disease Improving Global Outcomes (KDIGO). Interventional, control, and healthy subject groups were matched by sex and renal function. The eGFR was calculated with the abbreviated chronic kidney disease epidemiology formula (CKD-EPI), as defined by Levey et al. [11]. Nine patients were affected by autosomal dominant polycystic kidney disease, five patients with CKD had not a renal biopsy performed, one patient had Berger’s glomerulonephritis, and one patient had chronic pyelonephritis. Statins, antihypertensive, and antiplatelet therapies and/or therapies with calcium, calcitriol, and phosphate binders were continued in all patients included in the study.

### 2.3. Inclusion Criteria

Patients aged >18 and <80 years with CKD stage 3G–4G KDIGO (15 mL/min ≤ eGFR ≤ 60 mL/min) on conservative therapy were included. 

### 2.4. Exclusion Criteria

Patients were excluded who had severe heart disease (NYHA class IV) or acute heart failure; severe ongoing infections, congenital heart disease, neoplastic diseases, chronic liver disease, inflammatory bowel disease, and acute coronary syndrome within 3 months before the study initiated; patients who refused to give consent; and patients with missing data. 

### 2.5. Laboratory Measurements

In all patients, the levels of serum uric acid (SUA, mg/dL) and C-reactive protein (CRP, μg/L) were measured using standard automated techniques. Interleukin (IL)-1β, IL-6, tumor necrosis factor alpha (TNF-α), and plasma nicotinamide adenine dinucleotide phosphate (NADPH) oxidase (NOX2) were assessed using the following ELISA kits: human IL-1β, IL-6, and TNF-α (by RayBiotech, Peachtree Corners, GA, USA) and human NADPH and NOX2 (MyBiosource, San Diego, CA, USA). Arterial blood gas was measured using a blood gas analyzer (Nova Phox Plus C, 200 Prospect Street, Waltham, MA, USA).

### 2.6. Short Form (SF-36) Health Survey

The SF-36 Health Survey is a 36-item, patient-reported survey of patient health and it is a measure of health status. The original SF-36 stemmed from the Medical Outcome Study (MOS), which was conducted by the RAND Corporation. The SF-36 consists of eight scaled scores, which are the weighted sums of the questions in their section. Each scale is directly transformed into a 0–100 scale on the assumption that each question carries equal weight. The lower the score, the greater the disability. The eight sections are vitality, physical functioning, bodily pain, general health perceptions, physical role functioning, emotional role functioning, social role functioning, and mental health [12].

### 2.7. NGS Genome Microbiota Data Analysis

Fecal samples were collected using the Preanalytical Sample Processing (PSP) Stool Tubes (Stratec Molecular, Berlin, Germany). Biological samples were stored using 8 mL of DNA stabilizer contained in the PSP Stool Tubes. DNA was extracted using the PSP Stool Kit (Stratec Molecular, Berlin, Germany). In order to remove fecal cell debris, samples were lysed at 95 °C and centrifuged. Finally, DNA was purified using a spin column system and quantified using a NanoDrop spectrophotometer

Hypervariable regions V3–V4 of the 16s rDNA gene were sequenced using the Illumina MiSeq 2 × 300 bp sequencer. Forward and reverse reads were merged into amplicons using PEAR [13]. Using Trimmomatic [14], amplicons shorter than 450 bp and with a mean Phred score lower than 30 were discarded. Reads were chimaera-checked and clustered into operational taxonomic units (OTUs) using the QIIME 1.9.1 pipeline [15] and the SILVA database release 132 as a reference [16]. Low-frequency OTUs were removed [17] and statistical analysis was performed with R 3.4.4., using the phyloseq package. Differential abundance analysis among groups was performed using the DESeq2 package, as suggested by McMurdie et al. [18]. 

## 3. Results

### 3.1. Microbial Diversity and Taxonomic Composition Differed between CKD Patients and HC

**Figure 1:**
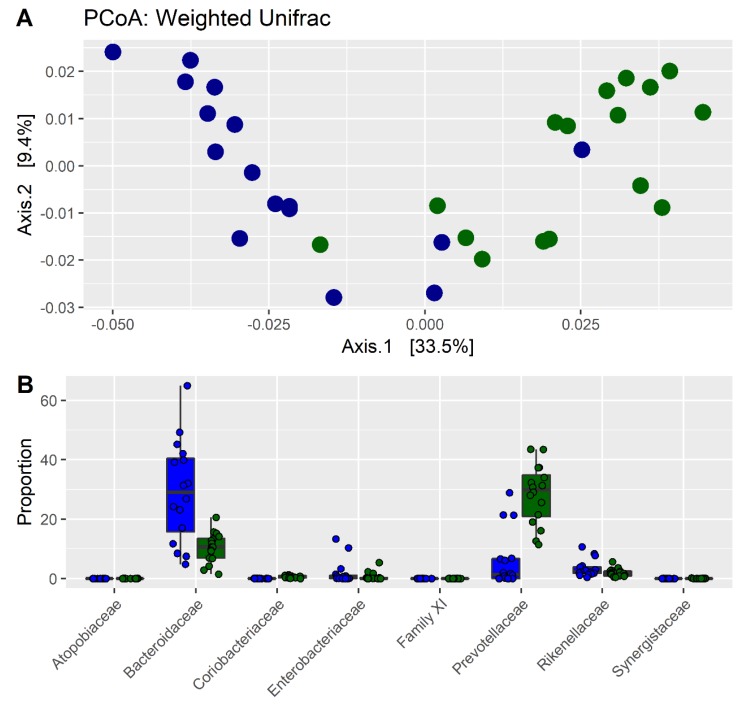
Microbial diversity and taxonomic composition in chronic kidney disease (CKD) patients and healthy control (HC) subjects: (**A**) principal coordinate analysis (PCoA) composition of taxonomic composition of fecal microbiota in CKD patients (blue dots) and controls (green dots), and (**B**) bacterial families frequencies in CKD patients (blue barplots) and controls (green barplots).

The intestinal microbiota of 16 CKD patients and 16 HC subjects was analyzed through the sequencing of the fecal 16s rRNA gene (V3–V4 regions) to identify a potential dysbiosis. The microbial diversity of fecal samples of CKD patients and the HC group was evaluated through principal coordinate analysis (PCoA) using the weighted UniFrac metrics. The PCoA plot indicates that the CKD and HC samples clustered into two different regions, suggesting that the gut microbiota of CKD patients is different from that of HC (Figure 1A). Significant differences in bacterial composition were also confirmed using the PERMANOVA test (*p* = 1 × 10^−4^; F = 1.74). The differential abundance indicates that eight bacterial families were significantly different between CKD patients and HC (Figure 1B, Supplementary Table S1). Gut microbiota of CKD patients contained higher levels of Bacteroidaceae, Enterobacteriaceae, and Rickenellaceae, while the HC group was characterized by higher levels of Atopobiaceae, Coriobacteriaceae, Clostridiales Family XI, Prevotellaceae, and Synergistaceae.

### 3.2. Effect of Dietary Intervention and Inulin Intake on Gut Microbiota in CKD Patients

**Figure 2:**
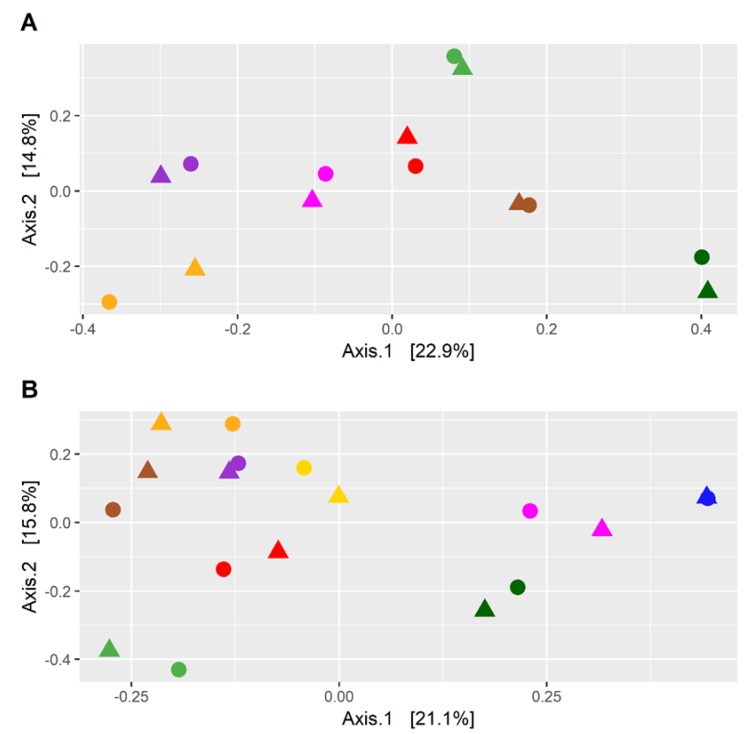
(**A**) PCoA plot of the fecal taxonomic composition of CKD patients before and after a 6 month dietary intervention. (**B**) PCoA plot of the fecal taxonomic composition of CKD patients before and after a 6 month dietary intervention comprising a low-protein diet (LPD) and intake of inulin. Samples collected before the dietary intervention are marked with a square, while samples collected after the dietary intervention are marked with a circle. Colors indicate the patients. We used weighted UniFrac metrics to compare the microbiota.

**Figure 3:**
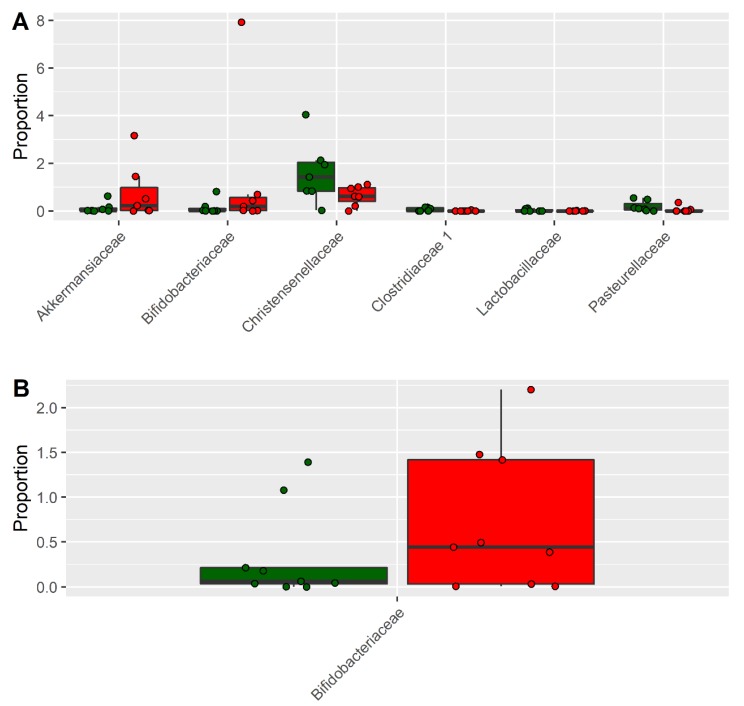
Taxa altered in CKD patients after 6 months of dietary intervention (**A**) or after 6 months of dietary intervention and intake of inulin (**B**). *Y*-axis shows the percentage of reads associated with bacteria families. Frequencies of bacteria before and after the intervention are reported in green and red, respectively.

**Figure 4:**
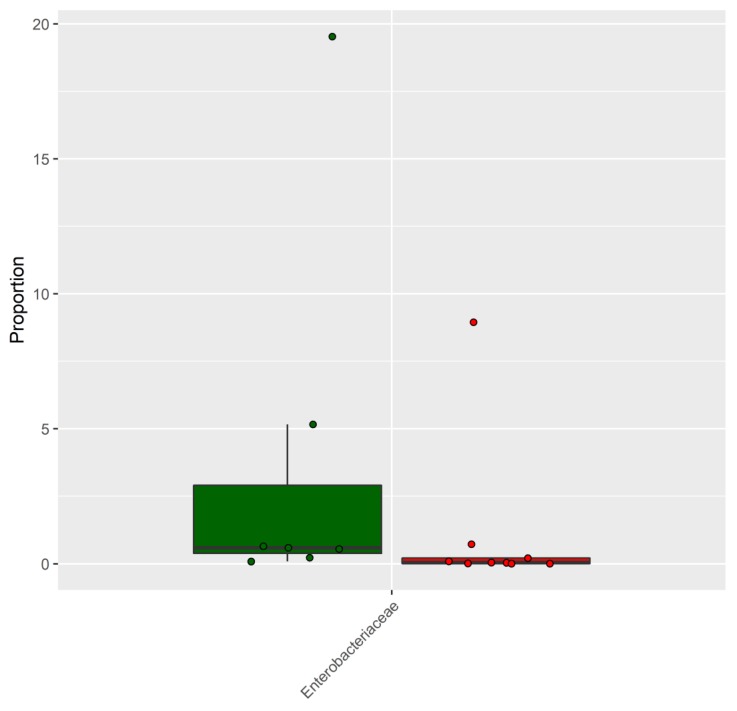
Taxa altered between CKD patients after 6 months of dietary intervention (green) or after 6 months of dietary intervention and intake of inulin (red).

The effect on the fecal microbiota of CKD patients before and after a dietary intervention of 6 months was also analyzed. In detail, nine patients underwent a dietary intervention with the supplement of inulin, while seven patients were controlled only by dietary intervention. The PCoA plot of the seven patients following only the LPD showed that the microbiota of each patient before and after the treatment was similar (Figure 2A), indicating that the dietary intervention had a small effect on the microbiota of each subject. This result was also confirmed by the PERMANOVA test (*p* = 0.96; F = 0.40). The analysis conducted on the nine patients following LPD and inulin led to similar results (Figure 2B) (*p* = 0.99; F = 0.29). However, despite the high similarity, a differential abundance analysis identified six bacterial families, the abundance of which was statistically different after the treatment (Figure 3A, Supplementary Table S2A). The LPD was able to significantly increase the frequencies of Akkermansiaceae and Bacteroidaceae and decrease the frequencies of Christensenellaceae, Clostridiaceae, Lactobacillaceae, and Pasteurellaceae. Only Bifidobacteriaceae were increased when the LPD was accompanied by oral inulin intake (Figure 3B, Supplementary Table S2B). Furthermore, the inulin intake led to a significant reduction of the Enterobacteriaceae family. The patients following LPD and inulin intake showed a lower frequency of Enterobacteriaceae (0.33%) compared to the patients following LPD only (0.71%) (Figure 4, Table S2).

### 3.3. Effect of Dietary Intervention and Inulin Intake on Clincal Parameters in CKD Patients

We found a significant reduction of SUA and CRP in patients treated with LPD and inulin (*p* = 0.018 and *p* = 0.003, respectively) but not in patients treated with LPD only, and there was a significant increase in serum bicarbonate both in patients treated only with LPD (HCO3^−^ at T0: 20.32 ± 2.42; HCO3^−^ at T1: 23.88 ± 2.81; *p* = 0.026) or with LPD and inulin (HCO3^−^ at T0: 22.17 ± 2.39; HCO3^−^ at T1: 25.01 ± 2.22; *p* = 0.01). We also observed a significant reduction in TNF-α (T0: 171.2 ± 90.3; T1: 116.2 ± 62.5; *p* = 0.041) and NOX2 (T0: 0.67± 0.1; T1: 0.58 ± 0.13; *p* = 0.027) in patients treated with LPD and inulin, while no changes were seen in terms of circulating IL-1β (*p* = 0.529) and IL-6 (*p* = 0.828) among the two groups. Also, we did not find a significant difference in eGFR at T1 between the two groups (*p* = 0.636). 

In parallel, among patients treated with LPD and inulin, we observed an improvement of the items “physical role functioning” and “general health perceptions” of SF-36 (*p* = 0.03 and *p* = 0.01, respectively).

## 4. Discussion

The intestinal microbiota performs various beneficial activities for the health of the host, in particular, regulating the absorption of nutrients, metabolism, the immune response, and the cellular signaling pathways, and it carries out protective activities against pathogenic bacteria and parasites [19]. These functions are mainly performed by saccharolytic bacteria, which are those that predominate in healthy conditions. These bacteria, by fermenting carbohydrates that have not been digested in the small intestine, are responsible for the production of various compounds, including short-chain fatty acids (SCFAs), which have numerous beneficial effects on the host, such as keeping the intestinal barrier intact, adjusting lipid and carbohydrate metabolism, regulating the immune system and the inflammatory and anti-inflammatory response, and reducing the pH of the intestinal lumen, which prevents the growth of pathogenic bacteria. However, different pathological conditions can lead to a dysbiosis, a term that indicates quantitative and qualitative alterations of the intestinal microbial composition and the metabolic activity of the microbiota. Among these, CKD is associated with intestinal dysbiosis and alteration of the intestinal barrier [20]. Although the mechanism through which intestinal dysbiosis occurs in CKD is still not entirely clear, it is hypothesized to be associated with several factors, such as dietary restrictions, frequent use of antibiotics, reduction of intestinal transit, alterations of the intestinal barrier, production of uremic toxins, and metabolic acidosis. In particular, in ESRD, the intestinal bacterial flora is significantly altered [21]. The most often reported changes in gut microbiota in CKD are related to the lower levels of Bifidobacteriaceae and Lactobacillaceae and higher levels of Enterobacteriaceae [6,22].

In our study, we found significant differences in the gut microbiota of CKD patients compared with HC. The gut microbiota of CKD patients contained higher levels of Bacteroidaceae, Enterobacteriaceae, and Rickenellaceae, while the HC was characterized by higher levels of Atopobiaceae, Coriobacteriaceae, Clostridiales Family XI, Prevotellaceae, and Synergistaceae. These alterations might be involved in the progression of CKD and in the increase of cardiovascular risk, uremic toxicity, inflammation, and oxidative stress [23]. Consequently, it appears necessary to reduce the intestinal dysbiosis by modulating bacterial growth with measures such as diet, prebiotics, and/or probiotics. In our study, we used an LPD coupled with or without the prebiotic inulin. Patients following only the LPD showed similar microbiota (Figure 2A), indicating that the dietary intervention had a small effect on the microbiota of each subject, perhaps due to the small sample size. However, the LPD was able to significantly increase the frequencies of Akkermansiaceae and Bacteroidaceae and decrease the frequencies of Christensenellaceae, Clostridiaceae, Lactobacillaceae, and Pasteurellaceae (Figure 3A). We found an increase of Bifidobacteriaceae when the LPD was accompanied by a regular intake of inulin (Figure 3B, Supplementary Table S2B). Bifidobacteria are symbiotic bacteria with a predominantly saccharolytic metabolism that determines the production of SCFAs, favoring adequate glycemic control and insulin resistance, an improvement in dyslipidemia, keeping the intestinal barrier intact, and reducing endotoxemia. Furthermore, the overgrowth of bifidobacteria inhibits the proliferation of pathogenic species, probably due to the reduction of colonic pH by the SCFAs. Li et al. [24] found a positive correlation between the beta diversity of gut microbiota and diet, suggesting that individuals with similar dietary habits harbor more similar gut microbiota. In our study, we found that in CKD patients, there was obvious dysbiosis of gut microbiota, including biased community constitutions (Figure 1A,B), as reported also by Xu et al. [25]. These observations may have important implications for treating dysbiosis, regulating the atypical gut microbiota by adjusting food species and structure and adding prebiotics. We showed a significant reduction of CRP and SUA only in patients treated with inulin and a significant increase of serum bicarbonate in both groups. Moreover, we found a significant reduction, only after treatment with inulin, in terms of TNF-α and NOX2 levels. Systemic inflammation, endothelial dysfunction, and oxidative stress play an important role in the progression of CKD and cardiovascular risk. There are many factors that contribute to the development of the inflammatory status in CKD, including increased production of proinflammatory cytokines, oxidative stress and metabolic acidosis, chronic and recurrent infections, altered metabolism of adipose tissue, and gut microbiota dysbiosis. Sources of inflammation, such as a dysregulation of gut microbiota or alteration of the intestinal barrier, can negatively impact the progression of CKD and uremia-associated complications [26]. The translocation of endotoxin and bacterial fragments into the subepithelial tissue leads to local inflammation via the activation of the resident immune system cells (macrophages, dendritic cells, and T cells), the release of proinflammatory cytokines and chemokines, and the infiltration of circulating inflammatory cells. Local production and the release of cytokines such as TNF-α, IL-6, and IL-1β could cause further disruption of intercellular tight junctions, increasing intercellular permeability. Moreover, CRP could act directly on the vascular endothelium, increasing the expression of adhesion molecules, reducing the expression of nitric oxide (NO) in endothelial cells, and altering low-density lipoprotein (LDL) [27,28]. We showed a significant reduction of SUA levels, which is considered a cardiovascular risk factor and it may promote endothelial dysfunction by increasing oxidative stress and thus favoring the development of atherosclerosis [29,30]. NOX are enzymes present in the cells of the vessel walls and physiologically produce reactive oxygen species (ROS) superoxide at low levels, but in pathological conditions, they produce cytotoxic levels of ROS, resulting in the initiation and progression of vascular disease, in particular, NOX2 isoforms. In fact, NOX2 knockout showed significantly reduced ROS levels, while the overexpression of NOX2 in mice caused a significant increase in superoxide production [31,32,33]. Therefore, increased activation of NOX2 could reduce NO production, promote oxidative stress, and, therefore, endothelial dysfunction favoring cardiovascular and renal damage in CKD patients [34,35]. We also found a significant improvement of metabolic acidosis in all the participants, confirming the importance of the LPD in CKD patients. In particular, the outcomes attributable to the LPD might also be mediated by the greater proportion of plant-based, base-producing food components. The base-producing food components in the LPD very likely mediated the increase in serum HCO3^−^. In addition, we can hypothesize that the improved serum HCO3^−^ likely contributed to the improvements in the SF-36 subjective measure of well-being and physical function, as recently demonstrated by others [36].

The correction of metabolic acidosis is essential, as it is a strong stimulus to protein and muscle catabolism, mineral metabolism diseases, insulin resistance, hyperkalemia, as well as being a known cardiovascular risk factor and a progression factor towards ESRD [37].

Our study has several limitations, including the small number of patients considered, which limits the interpretation of the results and does not allow for drawing definite conclusions. Therefore, additional prospective follow-up studies with a larger number of patients are necessary to confirm our observations. A significant proportion of patients were on several medications with a potential impact on different metabolic indices that may have possibly confounded the results. Moreover, the follow-up period was relatively short.

## 5. Conclusions

In conclusion, in the present study, we found a significant reduction in CRP, TNF-α, NOX2, and SUA levels, when patients were following a LPD coupled to inulin. Moreover, we found a significant improvement of metabolic acidosis and quality of life in the patients. These results suggest that various interventions attempting to modulate the gut microbiome may represent strategies to improve clinical outcomes in CKD patients and may provide useful therapeutic effects through the suppression of iNOS induction and improvements in the quality of life of CKD patients.

## Supplementary Materials

The following are available online at https://www.mdpi.com/2072-6643/11/12/3006/s1, Table S1: Bacterial families altered between CKD patients and Healthy Controls (HC), Table S2: Bacterial families altered in CKD patients before and after a dietary intervention.
