# Supplementary material for: Effect of Low-Protein Diet and Inulin on Microbiota and Clinical Parameters in Patients with Chronic Kidney Disease
Source: Nutrients. 2019 Dec 9;11(12):3006. doi: 10.3390/nu11123006 (PMC6950025; doi:10.3390/nu11123006)
Supplement: Supplementary file 1 [file nutrients-11-03006-s001.pdf]

Family	Pc	CKD	HC
Atopobioaceae	2E-03	9E-03	1E-02
Bacteroidaceae	5E-06	2E+01	1E+01
Coriobacteriaceae	5E-08	4E-02	6E-01
Enterobacteriaceae	4E-02	2E00	6E-01
Family XI	2E-03	3E-02	1E-02
Prevotellaceae	1E-02	6E00	2E+01
Rikenellaceae	9E-03	3E00	2E+01
Synergistaceae	6E-03	5E-02	3E-02

Table S1. Bacterial families altered between CKD patients and Healthy Controls (HC).Pc = adjusted p-value for multiple testing using DESeq2, Mb = mean read frequency associated to the Family before the treatment, Ma = mean read frequency associated to the Family after the treatment

	A) **Dietary intervenction**			B) **Dietary intervenction and intake of inulin**		
**Family**	Pc	Mb	Ma	Pc	Mb	Ma
Akkermansiaceae	2E-02	1E-01	7E-01	-	-	-
Bifidobacteriaceae	1E-02	1E-01	1E-01	8E-02	3E-01	7E-01
Christensenellaceae	3E-02	1E-01	6E-01	-	-	-
Clostridiaceae 1	1E-05	6E-02	7E-02	-	-	-
Lactobacillaceae	9E-02	3E-02	4E-02	-	-	-
Pasteurellaceae	2E-05	2E-01	6E-02	-	-	-

Table S2. Bacterial families altered in CKD patients before and after a dietary intervention (A) and dietary intervention and intake of inulin (B). Pc = adjusted p-value for multiple testing using DESeq2, Mb = mean read frequency associated to the Family before the treatment, Ma = mean read frequency associated to the Family after the treatment
